# In vitro antimicrobial efficacy of *Cassia alata* (Linn.) leaves, stem, and root extracts against cellulitis causative agent *Staphylococcus aureus*

**DOI:** 10.1186/s12906-023-03914-z

**Published:** 2023-03-18

**Authors:** Seng Chiew Toh, Samuel Lihan, Scholastica Ramih Bunya, Sui Sien Leong

**Affiliations:** 1 Department of Animal Science and Fishery, Faculty of Agricultural Science and Forestry, Universiti Putra Malaysia Bintulu Sarawak Campus, Nyabau Road, 97008 Bintulu, Sarawak Malaysia; 2 Institute of Biodiversity and Environmental Conservation, Universiti Malaysia Sarawak, 94300 Kota Samarahan, Sarawak Malaysia; 3 Faculty of Resource Science and Technology, Universiti Malaysia Sarawak, 94300 Kota Samarahan, Sarawak Malaysia; 4 Institute of Ecosystem Science Borneo, Universiti Putra Malaysia Bintulu Sarawak Campus, Nyabau Road, 97008 Bintulu, Sarawak Malaysia

**Keywords:** *Cassia alata*, Skin bacteria, Maceration and Soxhlet extraction, Antimicrobial, Phytochemicals, GC–MS

## Abstract

**Background:**

Cellulitis is a common skin disease encountered in medical emergencies in hospitals. It can be treated using a combination of antibiotics therapy; however, the causative agent *Staphylococcus aureus* has been reported to develop resistance towards the currently used antibiotics. Therefore, the search for more alternative herbal origin antimicrobial agents is critical. Aim: In this study, maceration and Soxhlet extraction of the whole plant of *Cassia alata* Linn. (leaves, roots, and stem) were performed using four solvents with different polarities, namely n-hexane, ethyl acetate, ethanol and distilled water. The crude extracts were screened using agar well diffusion, colorimetric broth microdilution, grid culture and bacterial growth curve analysis against *Staphylococcus aureus*. The phytochemicals in the crude extracts were identified using Gas Chromatography-Mass Spectrometry (GC–MS).

**Results:**

Agar-well diffusion analysis revealed that extraction using ethyl acetate showed the largest inhibition zone with an average diameter of 15.30 mm (root Soxhlet extract) followed by 14.70 mm (leaf Soxhlet extract) and 13.70 mm (root maceration extract). The lowest minimum inhibitory and minimum bactericidal concentration in root Soxhlet extract using ethyl acetate was 0.313 and 0.625 µg µL^−1^, respectively. Our study proved that crude extract of the plant suppressed the growth of *S. aureus* as evidenced from a significant regression extension (*p* < 0.06, *p* = 0.00003) of lag phase for 6 h after the treatment with increased concentration. Based on the GC–MS analysis, 88 phytochemicals consist of fatty acids, esters, alkanes, phenols, fatty alcohols, sesquiterpenoids and macrocycle that possibly contributed to the antimicrobial properties were identified, 32 of which were previously characterized for their antimicrobial, antioxidant, and anti-inflammatory activities.

**Conclusion:**

Ethyl acetate crude extract was better than the other investigated solvents. The root and stem of *C. alata* showed significant antimicrobial efficacy against *S. aureus* in this study. The remaining 56 out of 88 phytochemicals of the plant should be intensively studied for more medicinal uses.

**Supplementary Information:**

The online version contains supplementary material available at 10.1186/s12906-023-03914-z.

## Background

Cellulitis is commonly defined as an acute, spreading pyogenic inflammatory condition involving the dermis and subcutaneous tissues caused by bacterial infection [1–3]. It is, however; not contagious despite the rapid spreading and pyogenic nature of cellulitis [4]. Cellulitis is a general medical emergency in hospitals because it has caused more than 600,000 hospitalizations, about 3.7% of total emergency admissions in the US since 2010; the severity varies from mild to life-threatening [5, 6]. The Infectious Disease Society of America (IDSA) suggests that *Staphylococcus aureus* is the main culprit of cellulitis based on combined data from the studies of specimen cultures on punch biopsies, needle aspirates, and serological studies [7, 8].

*S. aureus* is a coagulase-positive, non-motile, and non-spore-forming facultative gram-positive anaerobe that colonize half of the adult population. Approximately 20 to 30% of them are permanent while the other 30% are just transient carriers [9]. It increases the risks of cellulitis by two to ten times [10]. When a staphylococcal infection is suspected or the aetiology that leads to cellulitis infection is unknown, patients are usually treated first with intravenous flucloxacillin and amoxicillin [11, 12]. In most cases, penicillin remains the first-choice drug for *S. aureus* infections [9]. However, it has been reported to develop resistance towards penicillin [13, 14], linezolid or daptomycin [15], glycopeptides, vancomycin, and teicoplanin [16] and significant resistance against the current generation antibiotics is anticipated in the near future.

The resistant of bacteria towards antimicrobial agents, increase in treatment costs and the adverse effects of synthetic drugs have necessitated the development of alternative, safe, efficient, and cost-effective natural medicines from plants [17, 18] and microbes [19–21]. Natural drugs are relatively cheaper; they have fewer side effects, better patient tolerance, and are acceptable due to a long history of use [22, 23]. The essential oils derived from medicinal plants are potential sources of antimicrobial agents against multiple-drug resistant (MDR) bacteria. These oils consist of phytochemicals such as terpenoids that can easily diffuse across cell membranes to induce biological reactions [24]. It reduces the opportunity for bacteria to develop resistance as the bacteria can be targeted via several mechanisms [25]. Besides, the essential oils also confer synergetic effects when used in combination with less effective antibiotics [26]. Hence, researchers are increasingly drawing their attention to medicinal plants for new leads to develop better solutions against MDR bacteria [27].

*Cassia alata* (Linn.) is known as the candle shrub by the locals in Sarawak, Borneo. It is a valuable plant, particularly among traditional practitioners in Malaysia. The locals use the plant as a prescriptive medicine to treat ringworm infection [22]. *C. alata* has been characterized by various bioactive compounds, including alkaloids, phenolics, flavonoids, tannin, steroids, and triterpenoids [28]. For example, kaempferol, anthraquinone, hexadecanoic acid methyl ester, hexadecanoic acid, and kaempferol-3-O-β-D-glucopyranoside were identified in leaves, while ziganein, apigenin, and 1,3,8-trihydroxy-2-methyl-anthraquinone were found in stem [28]. These phytochemicals possess exciting biological and pharmacological properties such as antimicrobial, antifungal [29, 30], antioxidants [31], antiseptic [32], anti-inflammatory [22], analgesic [33], and anti-hyperglycaemic [17, 34]. Previous studies have also shown the efficacy of the extracts and phytochemicals from *C. alata* against some clinical isolates of MDR bacteria [35, 36], which attracted researchers’ attention to explore the full potential of the plant as antibacterial and anti-oxidative agents. Hence, the main objective of this study was to determine the chemical compositions and antimicrobial properties of crude extracts from *C. alata* (Linn.) for potential application in the pharmaceutical sector against the cellulitis agent, *S. aureus*.

## Methods

### Plant material

Whole plants of *C. alata* Linn. were collected in June 2018 from a site located near Kampung Sungai Bako Jaya (N 1°40′25.5" E 110°27′12.8"), Malaysia. Botanical identification of the collected plant materials was done by Associate Professor Dr Mohd Said Saad of Plant Genetics Unit, Institute of Bioscience, Universiti Putra Malaysia. The voucher specimen was deposited at the Phyto-medicinal Herbarium of Institute Bioscience under the accession number SK179/02.

### Sample collection

*C. alata* plants (leaves, roots, and stems) were collected in sterile polyethene bags to avoid external contamination. The samples were labelled and transported directly to the laboratory in a chilled icebox.

### Sample processing

The leaves, roots, and stems were processed as described by Odeyemi et al*.* [37] with modifications. First, the samples were sorted according to appearance and condition, while those in a spoilt state were discarded. It was followed by surface disinfection of selected samples by soaking in 2% sodium hypochlorite (Merck, Germany, 6–14% active chlorine) for 10 min, followed by 70% ethanol (Merck, Germany, EMSURE® ACS) for a minute and then rinsed at least five times with sterile distilled water. The samples were then oven-dried at 40 °C for 72 h until a constant weight was obtained. After that, the dried samples were finely ground into small particle sizes (< 0.2 mm) and then transferred into sterile containers for storage in a dry condition.

### Maceration and Soxhlet extraction

Maceration was performed according to Yeo et al*.* [38] and Azwanida [39] with modifications. Four extraction solvents with different polarities were selected for the maceration process, namely n-hexane (Hex) (Merck, Germany, EMSURE® ACS), ethyl acetate (EA) (Merck, Germany, EMSURE® ACS), undenatured absolute ethanol (EtOH) (Systerm, Malaysia, ChemAR 99.8%), and sterile distilled water (dH_2_O). Approximately 20.0 g of ground samples were weighed and transferred to the screw-capped amber conical flasks. These samples were then soaked in the respective solvent in the ratio of 1 (sample): 10 (solvent) for 48 h and constantly mixed at room temperature on the platform shaker. After that, the sample-solvent mixtures were filtered and collected in sterile amber chemical bottles. A new batch of respective solvents was added to filtered samples for another round of extraction. These filtration and extraction processes were repeated four times to allow maximum recovery of bioactive compounds from the plant materials.

Soxhlet extraction was performed according to Redfern et al*.* [40] with modifications. Hex, EA, EtOH and dH_2_O were applied in the extraction process. Approximately 10.0 g of ground samples were weighed and transferred into cellulose extraction thimbles inside the Soxhlet extraction chamber. 200 mL of extraction solvent was added to the extractor flask and heated using an isomantle heater. These Soxhlet extractions were repeated for 20 cycles at 50 °C. After the extraction process, extraction solvents were collected in amber chemical bottles before rotary evaporation.

### Rotary evaporation

Crude extracts were dried in a rotary evaporator (Hei-VAP, Heidolph, Germany) to remove the excess of extraction solvents. Supplementary 1 lays the conditions of rotary evaporation for the respective extraction solvent. These crude extracts were rotary evaporated until a minute volume was left inside the flask. The leftover was then transferred into a pre-weighed sterile beaker and the flask was rinsed using a small volume of extraction solvent to allow maximum extract recovery. The beakers were dried at room temperature in the fume hood. These crude extracts were kept in a freezer at -20 °C for storage and further study.

### Solvent reconstitution

The extract colloids were reconstituted by using 100% (v/v) dimethyl sulfoxide (DMSO) (Merck, Germany, EMSURE® ACS). Several concentrations of plant extracts were calculated and prepared.

### Standardization of bacteria culture

A clinical strain of *S. aureus* was obtained from the Faculty of Medical and Health Science, Universiti Malaysia Sarawak. Unless stated otherwise, the antimicrobial assay was carried out using Mueller–Hinton broth (MHB) (Oxoid, UK). The bacterial culture was incubated at 37 °C for 18 h to obtain bacteria culture in the log phase and then standardized to 0.5 MacFarland at 600 nm.

### Agar well diffusion assay

The agar well diffusion assay was performed according to Magaldi et al*.* [41] and Valgas et al. [42] with slight modifications. Muller-Hinton agar (MHA) (Oxoid, UK) were equally divided into four different sections and labelled with types, extraction methods and concentrations of plant extract tested. Standardized *S. aureus* from overnight MHB culture was lawn cultured using a sterile cotton swab and then allowed to dry for 15 min. After that, 7 mm bores were punched through the seeded MHA and 50 μL of extracts with adjusted concentrations (1, 1.5, 2, 10 gL^−1^) were carefully transferred into the bores using a pipette. 50 μL of 100% (v/v) DMSO was used as the negative control. The agar plates were allowed to acclimate at room temperature for 15 min before being incubated at 37 °C for 18 h. The growth inhibition zones developed around the bores were measured in diameter using a pair of callipers. The assay was performed in triplicate, and the antimicrobial activities of the crude extracts were expressed as the mean of inhibition diameters in millimetres (mm).

### MIC using colorimetric broth microdilution assay

Broth microdilution assay was performed using sterile 96-wells round-bottom microtiter plates (TPP, Switzerland) according to Salvat et al*.* [43] and CLSI [44] standard with modifications. 100 μL of sterile MHB was dispensed using a multi-channel pipette into wells from rows B to H for columns 1 to 4, columns 6 to 9; rows A to H for columns 10 and 12. 200 μL of four prepared crude extracts with 10.0 gL^−1^ concentration were dispensed into row A, columns 1 to 4 for assays and columns 6 to 9 as the extract control. After that, 100 μL of crude extracts from row A were transferred and serially diluted to 2-fold from rows A to H for columns 1 to 4 and columns 6 to 9. It was followed by dispensing 200 μL of 100% (v/v) DMSO into the well in row A column 12 and serially diluted to 2-fold from rows A to H. For microbial inoculum, 0.5 MacFarland standardized *S. aureus* was further diluted 1:150 to obtain the bacteria concentration at 1 × 10^6^ CFU mL^−1^. Finally, 100 μL of the standardized bacteria inoculum was dispensed into the wells of rows A to H for columns 1 to 4 for assay and 12 for bacteria growth control. The same volume of sterile MHB was dispensed into wells from columns 6 to 9 for the extract control. The microtiter plates were read at 600 nm using an M965 microplate reader (Metertech Inc., Taiwan) after a minute of high-speed shaking before being incubated at 37 °C for 18 h.

After incubation, the plates were read again with the same set of conditions. Then, 20 µL of 0.45 µm syringe filtered 3-(4,5-dimethylthiazol-2-yl)-2,5- diphenyltetrazolium bromide (MTT) (AMRESCO, Ohio, USA, ultra-pure grade) solution (5.0 gL^−1^ in PBS) was subsequently added to all the 96-wells. The microtiter plates were wrapped with aluminium foil and incubated again at 37 °C for an hour. Purple formazan crystal was observed at the bottom of the wells shortly after the incubation. Three-quarters of the uncoloured MTT solution was carefully removed using a pipette without disturbing the formazan crystals. Next, the wells were washed using sterile PBS solution and the microtiter plates were placed on an orbital shaker at 120 rpm for three hours to settle down the formazan crystal. These processes were repeated twice to allow better of unbound MTT from the wells. After that, approximately 100 μL of formazan solubilizing agent (0.5% SDS, 36 mM HCL acidified isopropanol) was dispensed into all the 96-wells and mixed properly to dissolve all the formazan crystals. The microtiter plates were read at 540 nm after high-speed shaking for three minutes. The reduction of bacteria was calculated in percentage according to the formula shown below:

$$\mathrm{Percentage}\;\mathrm{of}\;\mathrm{bacteria}\;\mathrm{reduction}\;\left(\%\right)\;=\;\left[\frac{\left(Treated\;-\;blank\right)\;-\;\left(Control\;-\;blank\right)}{\left(Control\;-\;blank\right)}\right]\;\times\;100\%$$

### Minimum Bactericidal Concentration (MBC) using grid culture

MBC was performed under a standardized set of conditions as described in document M26-A [44]. The MBC was determined by subculturing from wells after broth microdilution assay to a non-selective agar, and negative microbial growth was yielded. Briefly, 3 µL of extract-bacteria mixture from the assay wells of broth microdilution assay after 18 h of incubation was pipetted onto the MHA plate that was gridded and labelled. Six extract concentrations were tested in triplicate: 5, 2.5, 1.25, 0.625, 0.313 and 0.156 µg µL^−1^. The inoculated MHA plates were acclimatised to room temperature for about 15 min before incubating at 37 °C for 18 h. The growth of *S. aureus* in the spaces was observed and the results were tabulated.

### Bacterial growth curve analysis

Bacterial growth curve analysis was performed according to Husain et al*.* [45] with modifications to determine the effects of crude extract on the bacterial growth curve of *S. aureus*. The analysis was conducted in the 96-wells round-bottom microtiter plates (TPP, Switzerland), and the absorbance was read using a microtiter plate reader. The identified MIC of the extracts against *S. aureus* from previous colorimetric broth microdilution assay was applied in this test. Sterile MHB containing the extracts was prepared at a final concentration of 0.5, 1 and 2 × MIC on a microtiter plate for testing and extract background control while sterile MHB without extract served as the blank and bacteria growth control. The *S. aureus* inoculum was prepared according to the previous broth microdilution assay. After that, one volume of standardized bacteria was added to the extract and bacteria growth control while a similar volume of sterile MHB was dispensed into extract background control and blank. The plates were incubated at 37 °C for varied time intervals (0, 2, 4, 6, 8, 12 and 18 h) and then read at 600 nm wavelength after a minute of high-speed shaking. Analysis was conducted in triplicate, and the bacterial growth curves expressed in the mean of turbidity where the absorbance was plotted against time intervals.

### Gas Chromatography-Mass Spectrometry (GC–MS)

GC–MS was performed as described by Samling et al*.* [46] with modifications. The analysis was conducted on Shimadzu GC–MS model QP 2010 PLUS (Shimadzu, Japan) equipped with a single quadrupoles mass analyser and a non-polar GC BPX-5 cross-linked column (5% Phenyl Polysilphenylene-siloxane) of 30 m length, 0.25 mm internal diameter and 0.25 µm film thickness. The temperature of the GC oven was initially programmed at 50 °C for 1 min then ramped to 240 °C at the rate of 8.5 °C min-1 and held for 10 min. Injector and detector temperatures were programmed at 260 °C. The interface temperature was set at 260 °C and the inert ion source was programmed at 200 °C while 70 eV of electron impact ionization energy was used with a scan rate and mass range of 909 s/spectra and 40–500 m/z. Helium gas (99.999% purity) was used as a carrier gas with a flow rate of 1.0 mL min-1. The injection volume was 1 µL with a splitting ratio of 20:1. The interpretation of mass-spectrum was conducted using a mass spectral library search in the National Institute Standard and Technology (NIST) database incorporated with the GC–MS data system for the potential identification of compounds. Name, molecular mass, and structure of the components in the crude extracts were analysed and recorded.

## Results

### Agar-well diffusion assay

**Fig. 1:**
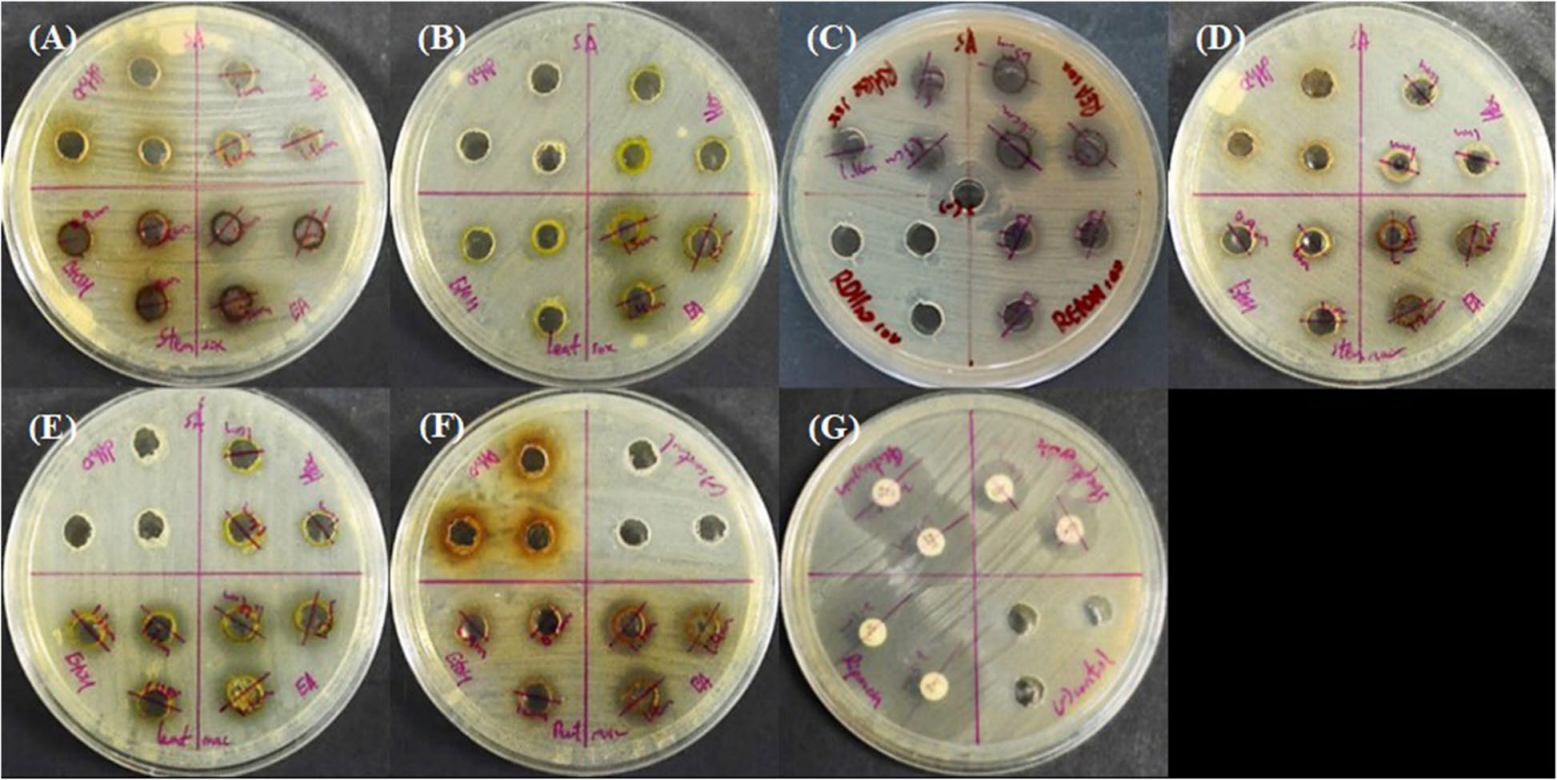
Agar well diffusion assay of the roots, stem, and leaves extract of *C. alata* from both maceration and Soxhlet extraction tested at 10 µg µL^−1^ concentration, where (**A**, **B**, **C**, **D**, **E**, **F**) represents Stem Soxhlet extract, Leaves Soxhlet extract, Root Soxhlet extract, Stem maceration extract, Leaves maceration extract, Root maceration extract, respectively. **G** represents Negative control-100% DMSO and positive controls-Rifampin, Gentamicin and Streptomycin antibiotic disks. Solvents used: Hex, n-hexane; EA, ethyl acetate; EtOH, undenatured ethanol & DH_2_O, sterile distilled water

Table 1 shows the average diameter of inhibition zones produced by *C. alata* crude extracts from both maceration and Soxhlet extraction tested in several concentrations with different plant’s parts and extraction solvents. The results showed that ethyl acetate extracts from both maceration and Soxhlet extraction generally exhibited stronger antimicrobial activities (*p* < 0.06, *p* = 0.0001) compared to the other extracts. The largest inhibition zone was observed in REA Soxhlet extract with an average diameter of 15.30 mm at 10 µg µL^−1^ extract concentration, followed by LEA Soxhlet (14.70 mm) and REA maceration (13.30 mm). These findings implied that ethyl acetate could be a better solvent than the others in isolating the phytochemicals responsible for antimicrobial activities towards *S. aureus* from *C. alata*. Generally, crude extracts of *C. alata* demonstrated a concentration-dependant antimicrobial activity because most of the extracts exhibited larger size of inhibition zones at higher concentrations and the sizes decreased with lower concentration. When the extract concentration was gradually reduced to 1 µg µL^−1^, there were only three extracts that remained susceptible towards *S. aureus* (*p* < 0.06, *p* = 0.01). Those extracts were SEA maceration extract (9.00 mm), REA maceration extract (9.70 mm) and REA Soxhlet extract (9.00 mm). Except for LdH_2_O Soxhlet extract at 10 µg µL^−1^ concentrations, most of the water extracts did not show clear inhibition zones. Figure 1 shows the agar well diffusion assay among leaves, roots, and stem parts of *C. alata* against *S. aureus* from both maceration and Soxhlet extraction at 10 µg µL^−1^ concentration.

**Table 1:** Average diameter of inhibition zones (mm) produced by *C. alata* crude extracts tested against *Staphylococcus aureus*

**Extraction**	**Parts**	**Solvent**	**Average diameter of inhibition zone (mm)**			
			**10 µg µL**^**−1**^	**2 µg µL**^**−1**^	**1.5 µg µL**^**−1**^	**1 µg µL**^**−1**^
Maceration	Stem parts	n-Hexane	11.7 ± 0.58	0.0	0.0	0.0
		Ethyl acetate	13.0 ± 0.00	9.3 ± 0.60	9.7 ± 0.60	9.0 ± 1.00
		Ethanol	10.7 ± 0.60	8.7 ± 0.60	8.3 ± 0.60	0.0
		dH2O	0.0	0.0	0.0	0.0
	Leaves	n-Hexane	10.3 ± 0.60	0.0	0.0	0.0
		Ethyl acetate	13.3 ± 0.60	9.0 ± 0.00	0.0	0.0
		Ethanol	13.3 ± 0.60	10.3 ± 0.60	0.0	0.0
		dH2O	0.0	0.0	0.0	0.0
	Root	n-Hexane	0.0	0.0	0.0	0.0
		Ethyl acetate	13.7 ± 0.60	9.3 ± 0.60	9.3 ± 0.60	9.7 ± 0.60
		Ethanol	9.3 ± 0.60	8.7 ± 0.60	9.3 ± 0.60	0.0
		dH2O	0.0	0.0	0.0	0.0
Soxhlet extraction	Stem parts	n-Hexane	10.3 ± 0.60	10.0 ± 0.00	0.0	0.0
		Ethyl acetate	13.3 ± 0.60	12.0 ± 0.00	10.0 ± 0.00	0.0
		Ethanol	8.3 ± 0.60	0.0	0.0	0.0
		dH2O	0.0	0.0	0.0	0.0
	Leaves	n-Hexane	0.0	0.0	0.0	0.0
		Ethyl acetate	14.7 ± 0.60	0.0	0.0	0.0
		Ethanol	11.3 ± 0.50	0.0	0.0	0.0
		dH2O	10.3 ± 0.60	0.0	0.0	0.0
	Root	n-Hexane	11.7 ± 1.20	0.0	0.0	0.0
		Ethyl acetate	15.3 ± 0.60	12.7 ± 0.60	10.7 ± 0.60	9.0 ± 0.00
		Ethanol	11.7 ± 0.60	9.3 ± 0.60	9.3 ± 0.60	0.0
		dH2O	0.0	0.0	0.0	0.0
Positive control- Rifampin antibiotic disc (5 µg)						26.0 ± 1.00
Positive control- Gentamicin antibiotic disc (10 µg)						22.0 ± 0.00
Positive control- Streptomycin antibiotic disc (10 µg)						14.5 ± 0.50
Negative control-100% (v/v) DMSO solution (50 µL)						0.0

### Minimum Inhibitory Concentration (MIC) and Minimum Bactericidal Concentration (MBC)

**Fig. 2:**
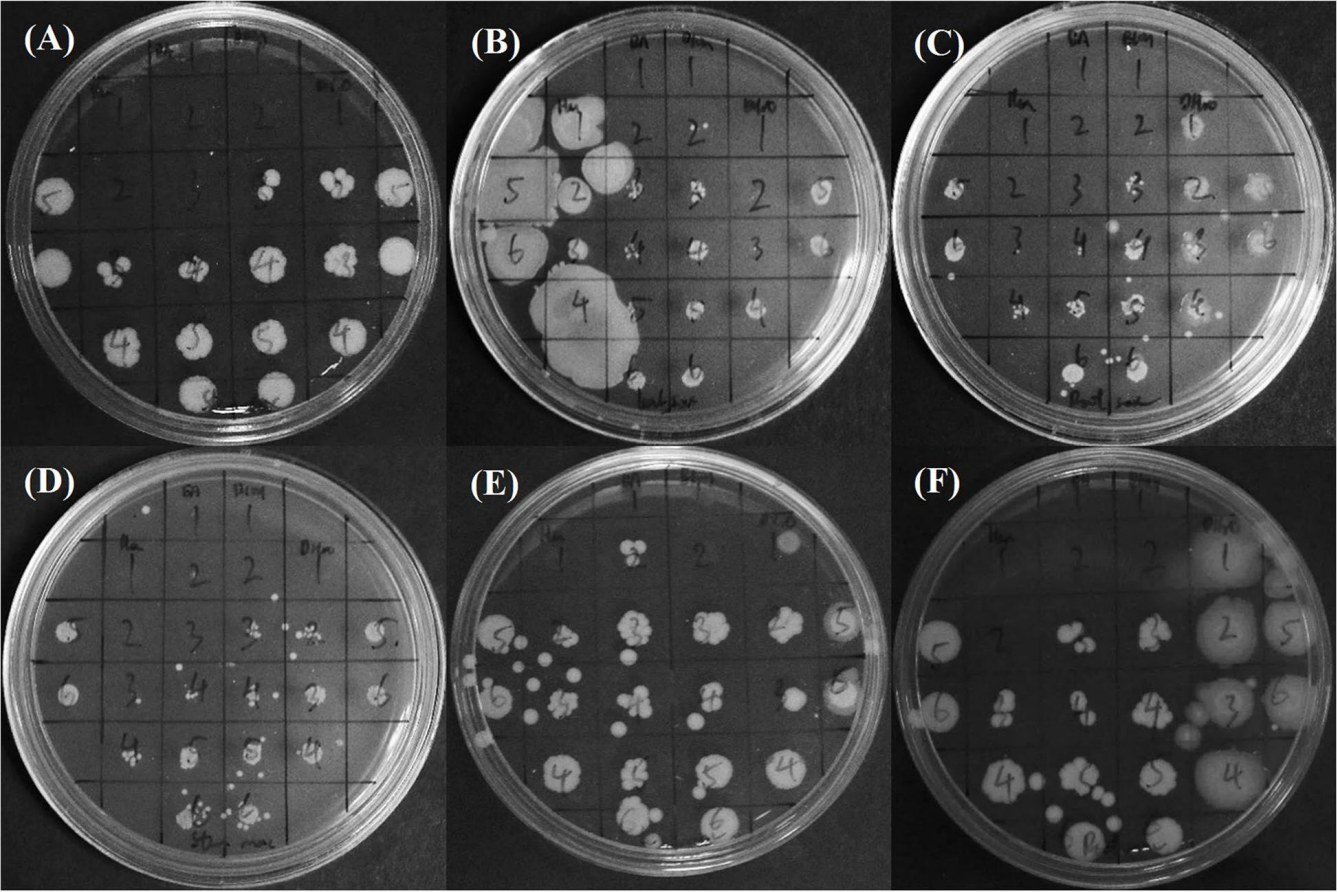
MBCs of crude extracts among stem parts, leaves and roots of *C. alata* from both maceration and Soxhlet extraction against *S. aureus*. Spaces without visible bacterial growth indicate bactericidal effect, where (**A**, **B**, **C**, **D**, **E**, **F**) represents Stem Soxhlet extract, Leaves Soxhlet extract, Root Soxhlet extract, Stem maceration extract, Leaves maceration extract, Root maceration extract, respectively

Table 2 shows the MIC, MBC endpoints and MBC/MIC ratio of crude extracts among leaves, roots and stem of *C. alata* from both maceration and Soxhlet extraction and the reduction percentage of bacteria identified at MIC endpoints. Figure 2 shows the overnight growth of *S. aureus* culture with the MBC values of the crude extracts against *S. aureus*. The maceration and Soxhlet extraction of stem, leaves and roots of *C. alata* using n-hexane, ethyl acetate and ethanol solvent produced most bactericidal agents (MBC/MIC ≤ 4), except for LEA maceration (MBC/MIC = 8) and LHex Soxhlet (MBC/MIC > 4) that are bacteriostatic agents. From the colorimetric broth microdilution assay, ethyl acetate extracts of *C. alata* with MIC endpoints ranging from 0.313 to 0.625 µg µL^−1^ showed strong antagonistic activities (*p* < 0.06, *p* = 0.00008) compared to the other extracts. Among the extracts tested, REA Soxhlet extract had the lowest MIC value, with 0.313 µg µL^−1^, while the other extracts ranged between 0.625 µg µL^−1^ to 1.25 µg µL^−1^. Almost all water extracts tested did not show any inhibition activity except for LdH_2_O Soxhlet extract at 1.25 µg µL^−1^ extract concentration. The REA soxhlet extract had exhibited the lowest MBC value with 0.625 µg µL^−1^ while the other extracts ranged between 1.25 to more than 5.00 µg µL^−1^. Almost all water-based crude extracts had higher MBC values, exceeding 5.00 µg µL^−1^.

**Table 2:** Minimum Inhibitory Concentration and Minimum Bactericidal Concentration of *C. alata* against *Staphylococcus aureus*

**Extraction**	**Parts**	**Solvent**	**Bacteria Reduction Percentage**	**MIC [µg/µL]**	**MBC [µg/µL]**	**MBC/MIC**
Maceration	Stem parts	n-Hexane	-92.954 ± 11.285	0.625	2.500	4
		Ethyl acetate	-99.082 ± 0.933	0.625	1.250	2
		Ethanol	-101.520 ± 4.825	1.250	2.500	2
		dH2O	-	-	5.000	-
	Leaves	n-Hexane	-99.327 ± 1.009	1.250	5.000	4
		Ethyl acetate	-91.743 ± 5.265	0.625	5.000	8
		Ethanol	-100.337 ± 2.554	1.250	2.500	2
		dH2O	-	-	5.000	-
	Root	n-Hexane	-99.760 ± 1.397	1.250	2.500	2
		Ethyl acetate	-98.740 ± 1.004	0.625	2.500	4
		Ethanol	-93.020 ± 11.951	1.250	2.500	2
		dH2O	-	-	> 5.000	-
Soxhlet extraction	Stem parts	n-Hexane	-90.910 ± 8.376	0.625	2.500	4
		Ethyl acetate	-97.219 ± 1.872	0.625	1.250	2
		Ethanol	-99.424 ± 1.771	1.250	1.250	1
		dH2O	-	-	5.000	-
	Leaves	n-Hexane	-99.618 ± 6.884	1.250	> 5.000	> 4
		Ethyl acetate	-98.810 ± 2.259	0.625	2.500	4
		Ethanol	-96.136 ± 6.214	1.250	2.500	2
		dH2O	-99.867 ± 0.992	1.250	1.250	1
	Root	n-Hexane	-99.031 ± 0.619	0.625	2.500	4
		Ethyl acetate	-95.937 ± 2.975	0.313	0.625	2
		Ethanol	-97.745 ± 1.848	1.250	2.500	2
		dH2O	-	-	> 5.000	-

### Bacterial growth curve analysis

**Fig. 3:**
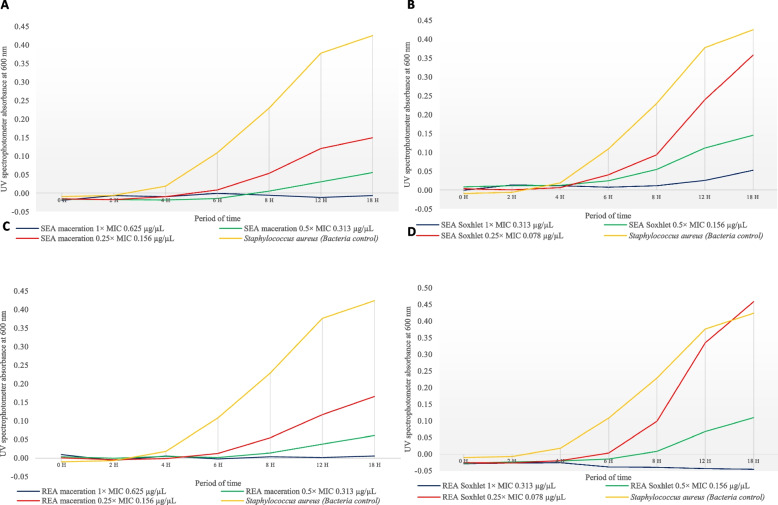
Growth curves of *S. aureus* treated with several MICs of SEA maceration, SEA Soxhlet, REA maceration and REA Soxhlet extract at 600 nm wavelength across 18 h of incubation where (**A**, **B**, **C**, **D**) represents SEA maceration, SEA Soxhlet, REA maceration, REA Soxhlet, respectively. Solvents used: EA, ethyl acetate. S and R represents stem and root

Further analysis of bacterial growth curve was performed for REA Soxhlet, SEA maceration, SEA Soxhlet, and REA maceration extracts that showed a large inhibition zone with low MIC and MBC endpoints and ratios. Figure 3 shows the growth curves of *S. aureus* treated with several MICs of REA Soxhlet, SEA maceration, SEA Soxhlet and REA maceration extract at 600 nm wavelength across 18 h of incubation. From the growth curves, the untreated *S. aureus* (orange lines, Fig. 3) managed to reach the log phase after 2 to 4 h of incubation at 37 °C. It started to grow exponentially afterwards and recorded an optical density (OD) of 0.424 after 18 h of incubation. However, the treated *S. aureus* generally recorded a proportional extension of lag phase for about 2 to 6 h and reduction in bacterial growth rate after the extract treatment with increasing extract concentration. At 0.25 × MIC concentration (red lines, Fig. 3), the SEA maceration, REA maceration and REA Soxhlet extracts-treated *S. aureus* showed a minor increment after 4 h of incubation, while those treated with SEA Soxhlet had a major increment in OD within a similar incubation. Meanwhile, *S. aureus* treated with the 1 × MIC extract concentration (blue lines, Fig. 3) of REA Soxhlet, SEA maceration and SEA Soxhlet extract only showed slight or no increment after 8 h of incubation, while those treated with REA Soxhlet extract had an OD decline after 4 h. The regression study between extract concentration and the optical densities of treated *S. aureus* was significant (*p* < 0.06, *p* = 0.00003). The increase in OD indicates that the crude extracts were unable to further induce the bacteriostatic or bactericidal effects due to depleting antimicrobial phytochemicals in the extracts, confirming the concentration dependency.

### Gas Chromatography-Mass Spectrometry (GC–MS)

REA Soxhlet, SEA maceration, SEA Soxhlet, and REA maceration extract were subjected to GC–MS analysis to identify the chemical constituents of the extracts. A total number of 88 individual phytochemical compounds were identified and 32 of them were readily known from previous studies to have antimicrobial, antioxidant, or anti-inflammatory properties. The individual compounds were identified based on mass spectra fragmentation patterns using the NIST14 library database (Table 3).

**Table 3:** Phytochemicals identified in *C. alata* (stem, leaves, roots) extracts that potentially use to treat cellulitis

**Phytochemicals**	**Extracts**	**R.T/min**	**Area (%)**	**Molecular Formula**	**M. W**	**Nature**	**Biological functions**	**References**
n-Hexadecanoic acid	SEA maceration SEA Soxhlet REA Soxhlet	15.094 15.120 15.195	12.28 28.79 34.34	C_16_H_32_O_2_	256	Saturated long-chain fatty acid	Antibacterial, anti-inflammatory, antioxidant, antiandrogenic, 5α-reductase inhibitor Shows pesticidal, nematocidal, haemolytic, hypocholesterolemic effects	[47–50]
9, 12-Octadecadienoic acid (Z, Z)-	SEA maceration SEA Soxhlet REA Soxhlet	16.233 16.261 16.341	11.65 38.00 10.07	C_18_H_32_O_2_	280	Unsaturated fatty acid (plant glycosides)	Nematicide, antiacne, insectifuge, antiarthritic, hepatoprotective and cancer-preventive, antiandrogenic, antieczemic, antihistaminic, anti-inflammatory, anti-coronary, 5α-reductase inhibitor, and shows hypocholesterolemic effects	[51–53]
Stigmasterol	SEA maceration	18.760	5.72	C_29_H_48_O	412	Phytosterol	Show strong antibacterial activity against multiple-drug resistant mycobacteria. Strong antioxidant, anti-inflammatory, antioxidative properties that can inhibit tumour promotion	[54–57]
γ-Sitosterol	SEA maceration	20.849	5.03	C_29_H_50_O	414	Plant steroid	Prophylactic activity, antioxidant, antibacterial, anti-diarrhoeal, and anti-inflammatory	[56, 58–60]
β-Sitosterol acetate	SEA maceration	26.416	3.95	C_31_H_52_O_2_	456	Steroid ester	Antioxidant and free radical scavengers	[55]
Octadecanoic acid	SEA maceration SEA Soxhlet REA Soxhlet	16.372 16.388 16.466	3.01 6.68 3.16	C_18_H_36_O_2_	284	C18 straight-chain saturated fatty acid	Antibacterial, antifungal, and antitumor activities	[52, 61]
Eicosanoic acid	SEA maceration	12.130 13.681	1.07 0.62	C_20_H_40_O_2_	312	C20 straight-chain saturated fatty acid	Anticancer and anti-inflammatory activities	[55, 62]
Octadecane	SEA maceration	12.367	1.14	C_18_H_38_	254	C18 straight-chain alkane	Antimicrobial, antioxidant, anticancer, and hypoglycaemic activities	[61, 63]
Dibutyl phthalate	SEA maceration	15.165	0.99	C_16_H_22_O_4_	278	Phthalate ester and a diester	Antimicrobial, antimalarial, and antifungal effects	[56, 61]
Phenol, 3, 5-bis (1, 1-dimethylethyl)-	SEA maceration	11.757	0.76	C_14_H_22_O	206	Alkylated phenol	Anti-inflammatory effects	[64]
1-Eicosanol	SEA maceration SEA Soxhlet	17.126 17.127	0.73 0.78	C_20_H_42_O	298	Straight-chain fatty alcohol	Antimalarial, antifungal and antioxidant	[65, 66]
n-Nonadecanol-1	SEA maceration	13.850	0.64	C_19_H_40_O	284	Long-chain fatty alcohol	Antimicrobial and cytotoxic effects	[61]
Tetradecane	SEA maceration	10.668	0.63	C_14_H_29_Cl	232	C14 straight-chain acyclic alkane	Antifungal, antiviral, antibacterial, nematocidal, antitumor and wound healing activities	[61, 67, 68]
Pentadecanoic acid	SEA maceration SEA Soxhlet	14.400 14.440	0.55 0.92	C_15_H_30_O2	242	C15 straight-chain saturated fatty acid	Antibacterial activities	[61, 68, 69]
1,2-Benzene dicarboxylic acid, bis(2-methylpropyl) ester	SEA maceration	14.494	0.53	C_16_H_22_O_4_	278	Phthalate ester and a diester	Antimicrobial, antifungal, and antifouling activities	[49, 61, 70]
Bicyclo[3.1.1]heptan-3-ol,6,6-dimethyl-2-methylene-,[1S-(1α, 3α, 5α)]	SEA maceration	17.388	0.45	C_10_H_16_O	152	Pinane monoterpenoid, Secondary alcohol, Carbobicyclic compound	Antimicrobial activities	[60]
Neophytadiene	SEA maceration REA maceration REA Soxhlet	14.153 14.169 14.170	0.44 1.98 3.57	C_20_H_38_	278	Sesquiterpenoid	A good analgesic and induce anti-inflammatory, antimicrobial, and antioxidant effect	[57, 71, 72]
Tetratetracontane	SEA maceration	15.898	0.43	C_44_H_90_	618	C44 long-chain unbranched alkane	Anti-inflammatory, antibacterial, antioxidant, antiulcerogenic and hypoglycaemic effects	[48, 61, 68]
Cycloheptasiloxane, tetradecamethyl-	SEA maceration	10.883	0.43	C_14_H_42_O_7_Si_7_	518	Organosiloxane and a macrocycle	Antioxidant, antimicrobial and hypocholesterolemic effects	[73]
Tetracontane	SEA Soxhlet	19.047	8.86	C_40_H_82_	562	Straight-chain aliphatic alkane	Analgesic and anti-inflammatory activity	[61, 69]
Palmitoleic acid	SEA Soxhlet	14.976	0.99	C_16_H_30_O_2_	254	Long chain minor monounsaturated fatty acid	Inhibit bacterial enoyl-acyl carrier protein reductase (FabI) and increase the permeability of bacterial membrane because of surfactant action	[74]
Tetradecanoic acid	SEA Soxhlet REA Soxhlet	13.681 13.714	0.85 0.58	C_14_H_28_O_2_	228	Long-chain saturated fatty acid	Nematicide and cancer-preventive agent, antimicrobial, antioxidant, antifungal, hypercholesterolemic effects	[51, 61, 70, 75]
Heptadecanoic acid	SEA Soxhlet	15.746	0.59	C_17_H_34_O_2_	270	C17 long, straight-chain saturated fatty acid	Antimicrobial, antioxidant, and antifungal Used as surfactant	[51, 61, 76]
9,10-Anthracenedione, 1, 8-dihydroxy-3-methyl-	SEA Soxhlet	18.037	0.58	C_15_H_10_O_4_	254	Trihydroxyanthraquinone	Antiviral and anti-inflammatory effects	[53]
Hexadecanoic acid, methyl ester	REA maceration REA Soxhlet	14.826 14.825	22.18 5.44	C_17_H_34_O_2_	270	Fatty acid methyl ester	Antifungal, antioxidant, 5α-reductase inhibitor, nematicide, pesticide, antiandrogenic, flavour hypocholesterolemic, haemolytic effects and potent antimicrobial activities	[48, 61, 68–70, 77]
9-Octadecenoic acid (Z)-, methyl ester	REA maceration REA Soxhlet	16.021 15.991	8.56 5.12	C_19_H_36_O_2_	296	Fatty acid methyl ester derived from oleic acid	Antioxidant, antimicrobial and nematocidal, anticarcinogenic, antihypertensive	[67, 77, 78]
Cyclohexasiloxane, dodecamethyl-	REA maceration	9.415	2.12	C_12_H_36_O_6_Si_6_	444	Organosiloxane and a macrocycle	Antifungal, antimicrobial, and used as a preservative and health-related products	[73, 79]
Phytol	REA Soxhlet	16.059	6.66	C_20_H_40_O	296	Acyclic diterpene alcohol	Antimicrobial, antioxidant, anti-inflammatory and anticancer It can be used as a precursor for the manufacture of synthetic forms of vitamin E and vitamin K1	[50, 51, 57, 77, 80]
Squalene	REA Soxhlet	21.158	1.43	C_30_H_50_	410	Natural isoprenoid	Precursor of various hormones in animals and sterols in plants. Cancer and chemopreventive agent, antibacterial, antioxidant, antitumor, immunostimulant, a lipoxygenase-inhibitor, and free radical scavenger	[48, 49, 51, 57, 61, 68]
3, 7, 11, 15-Tetramethyl-2-hexadecen-1-ol	REA Soxhlet	14.481	1.29	C_20_H_40_O	296	Acyclic diterpene alcohol	Insecticidal, anti-inflammatory, anti-tuberculosis, antimicrobial, and antioxidant	[57, 61, 62]
n-Heptadecanol-1	REA Soxhlet	17.152	0.86	C_17_H_36_O	256	Long-chain fatty alcohol	Anti-arthritis and treatment of skin diseases	[61]
2-Pentadecanone, 6, 10, 14-trimethyl-	REA Soxhlet	14.253	0.57	C_18_H_36_O	268	Sesquiterpenoid	Antibacterial activity against both gram-positive and gram-negative bacteria	[52, 61, 67, 70]

The SEA maceration had 50 major phytochemicals identified from the GC–MS, however, only 19 were previously characterized. Among the 19 phytochemicals, SEA maceration extract shared six phytochemicals in common and the remaining were found only specialized to the extract. N-hexadecanoic acid (12.28%), 9, 12-octadecadienoic acid (Z, Z)- (11.65%), octadecanoic acid (3.01%), 1-eicosanol (0.73%), pentadecanoic acid (0.55%) and neophytadiene (0.44%) were common, while stigmasterol (5.72%), γ-sitosterol (5.03%), β-sitosterol acetate (3.95%), eicosanoic acid (1.07%), octadecane (1.14%), dibutyl phthalate (0.99%), phenol, 3, 5-bis (1, 1-dimethylethyl)- (0.76%), n-nonadecanol-1 (0.64%), tetradecane (0.63%), 1,2-benzene dicarboxylic acid, bis(2-methylpropyl) ester (0.53%), bicyclo[3.1.1]heptan-3-ol,6,6-dimethyl-2-methylene-,[1S-(1α, 3α, 5α)] (0.45%), tetratetracontane (0.43%) and cycloheptasiloxane, tetradecamethyl- (0.43%) were found only in SEA maceration extract.

SEA Soxhlet had 17 major phytochemicals identified, however, only ten were previously characterized. Six were in common, namely 9, 12-octadecadienoic acid (Z, Z)- (38%), n-hexadecanoic acid (28.79%), octadecanoic acid (6.68%), pentadecanoic acid (0.92%), tetradecanoic acid (0.85%) and 1-eicosanol (0.78%). Meanwhile, tetracontane (8.86%), palmitoleic acid (0.99%), heptadecanoic acid (0.59%), 9,10-anthracenedione, 1, 8-dihydroxy-3-methyl- (0.58%) were the four phytochemicals that were found only in SEA Soxhlet extract.

Of the 17 phytochemicals identified in REA maceration from the GC–MS, only four were previously characterized, namely hexadecanoic acid, methyl ester (22.18%), 9-octadecenoic acid (Z)-, methyl ester (8.56%) and neophytadiene (1.98%). Cyclohexasiloxane, dodecamethyl- (2.12%) was the only phytochemical found explicitly to REA maceration.

There were 30 phytochemicals identified in REA Soxhlet, 12 of which were previously characterized and seven phytochemicals were found in common [n-hexadecanoic acid (34.34%), 9, 12-octadecadienoic acid (Z, Z)- (10.07%), hexadecanoic acid, methyl ester (5.44%), 9-octadecenoic acid (Z)-, methyl ester (5.12%), neophytadiene (3.57%), octadecanoic acid (3.16%), tetradecanoic acid (0.58%)]. Meanwhile, phytol (6.66%), squalene (1.43%), 3, 7, 11, 15-tetramethyl-2-hexadecen-1-ol (1.29%), n-heptadecanol-1 (0.86%) and 2-pentadecanone, 6, 10, 14-trimethyl- (0.57%) were the five explicit phytochemicals found in the REA Soxhlet extract.

## Discussion

Previous research has shown that the ethanolic and methanolic extracts of *C. alata* had stronger antimicrobial activities against *S. aureus* in agar-well or agar-disc diffusion assay compared to chloroform and ethyl acetate extracts [81, 82]. These differences in antimicrobial activities from different solvents and plant parts were also observed elsewhere [82, 83]. Hence, the present study expects significant antimicrobial activities from ethanol extracts. However, it was the extracts from ethyl acetate exhibited stronger antimicrobial activities. It implies that ethyl acetate may be a better solvent than others in extracting the phytochemicals responsible for antimicrobial activities against *S. aureus* from *C. alata*. Ethyl acetate is capable of extracting and dissolving active principal phytochemicals with semi-polar properties from plants, such as alkaloids, sterols, terpenoids, flavonoids, aglycons and glycosides [84]. The phytochemicals may be more soluble and potent when extracted using ethyl acetate compared to the other solvents [85]. Therefore, stronger antimicrobial activities demonstrated by ethyl acetate extract compared with other extraction solvents may be attributed to the presence of impurities or ashes that are more soluble in other solvents due to dilution effect of the active phytochemicals in the extracts [85].

The crude extracts of *C. alata* also demonstrated a concentration-dependant antimicrobial activity. These findings were in line with previously reported findings [85–87]. When the extract concentration was gradually reduced to 1 µg µL^−1^, only three extracts remained susceptible to *S. aureus* (*p* < 0.06, *p* = 0.01). Previous research has determined that the phytochemical distribution within *C. alata* varied between plant parts [82, 83, 85]. For example, the roots may accumulate more flavonoid quercetin, naringenin and kaempferol [42], while anthraquinones, flavonoids, quinines, and sterols in leaves [88]. This could explain why ethyl acetate root extract is more potent and exhibited stronger antimicrobial activities against *S. aureus* at all concentration levels compared to the leaves and stem extracts of same solvent [85].

On the contrary, most of the water extracts did not show clear inhibition zones at 10 µg µL^−1^ concentrations except for the LdH_2_O Soxhlet extract. These findings suggest that water extracts of *C. alata* did not possess any antimicrobial properties or inhibition effects against *S. aureus* [22]. Interestingly, previous research by Somchit et al*.* [30] and Timothy et al*.* [87] revealed the presence of certain phytochemicals in LdH_2_O Soxhlet extract that could inhibit the growth of *S. aureus*. However, findings from the present study contradict the indication that water extracts of *C. alata* shared the same potency as the other extracts tested in this study [89, 90]. The differences observed in antimicrobial activities of extracts from the same plant part tested are common in phytochemical research. This is because the concentration of plant constituents may vary from one geographical location to another, depending on the age of the plant, differences in topographical factors, soil nutrients, extraction methods as well as the method used for antimicrobial study [91].

From broth microdilution assay, the ethyl acetate extracts of *C. alata* demonstrated strong antagonistic activities. Similar findings were also reported by Wikaningtyas and Sukandar [92, 93], indicating that the ethyl acetate extracts could exhibit stronger antimicrobial activities against *S. aureus* compared to the other extracts of *C. alata*. The bacteria reduction rates of *S. aureus* at the MIC endpoints were higher than 90%, thereby, validating the MIC endpoint values of those extracts. For crude extracts with a bacteria reduction rate above 100%, the actual MIC endpoint values could be slightly higher than the values stated but that intermediate concentration was not determined. These findings substantiated the preliminary results from agar-well diffusion assay that most water extracts either only showed inhibition effects at very high concentrations or no inhibition effects at all towards *S. aureus*. However, these results contradicted previous findings where water extracts of *C. alata* were shown to be capable of inhibiting the growth of *S. aureus* and were just as effective as the other extracts [87, 91]. These differences in the antimicrobial activities could be attributed to variations in phytochemical concentrations that were active and potent against *S. aureus*, as well as water-soluble impurities from the extraction process [85, 91].

The extracts are only considered as bactericidal agents if the ratio MBC/MIC is less than or equal to 4, while those greater than 4 as bacteriostatic agent [89, 94]. The MBC/MIC ratio of crude extracts ranged between 1 and 4, except for LEA maceration extract with the ratio of 8. The MBC/MIC ratios of water-based extracts cannot be calculated due to the ambiguous values of MIC and MBC (except for LdH_2_O Soxhlet), indicating that water-based extracts possessed no or weak antimicrobial effects against *S. aureus*. Therefore, it can be inferred that most of the *C. alata* crude extracts could be utilised as the bactericidal agent while LEA maceration and LHex soxhlet as the bacteriostatic agent.

Bacterial growth curve analysis is an influential tool that can provide comprehensive information about the pharmacodynamics of an antibacterial agent that cannot be obtained simply through the endpoints assay such as the MIC and MBC [90, 95]. The primary focus of the analysis is the elongation of the lag phase, which is the time required for the bacterial culture to enter the exponential phase after the extract treatment, because the length of the lag phase directly depends on the bacteria’s growing condition [96]. The lag phase is also a critical window to protect the bacteria from antimicrobial stress and promote bacteria regrowth after the removal of antimicrobial agents [97]. Theophel et al*.* [98] emphasize the importance of the lag phase as the key stage for bacteria to develop strategies in resisting the killing by antimicrobial agents [98]. The researchers further highlighted the duration of lag phases as a more meaningful indicator of dose-dependent antibiotic inhibition. Hence, an in-depth understanding of how antimicrobial agents affect the lag phase of bacterium is essential.

In this study, *S. aureus* treated with *C. alata* extracts showed differences in the growth rate at the exponential phase (Fig. 3). For example, *S. aureus* treated with 0.25 × MIC concentration of SEA maceration extract showed an approximately 54.4% decrease in growth rate at 8 to 12 h of incubation compared with the bacteria control. At the same time, those treated with 0.5 × MIC concentration of SEA maceration extract showed approximately 93.2% of reduction for the same incubating period. Similar observations were also noted in other extracts with the increase of extract concentration. Meanwhile, *S. aureus* treated with REA Soxhlet extract at 0.25 × MIC concentration recorded higher optical density values after 18 h of incubation compared with the bacteria control. These observations could be due to the low inoculum concentration of REA Soxhlet extract (0.078 µg µL^−1^) and the promoting effect of bacterial regrowth upon the removal of antimicrobial agents after the extended lag phases [48, 99]. Based on the bacterial growth curve analysis, the period required for the extracts to show full potential efficacy against *S. aureus* was determined as 6 h of incubation. Hence, we suggest that *S. aureus* be treated with another concentration level at the 6^th^ hour to maintain low OD readings.

There were 88 individual phytochemical compounds identified from the GC–MS analysis and 32 were readily known from previous research to have antimicrobial, antioxidant, or anti-inflammatory properties. According to the chromatogram, the largest peak area (38.00%) was recorded by 9, 12-octadecadienoic acid (Z, Z)- (linoleic acid) in SEA Soxhlet extract. It is a plant glycoside with anti-inflammatory, antieczemic and inhibitory action against some bacteria species [51, 74]. However, despite the bacterial inhibitory action, most research effort has primarily focused on its anti-inflammatory properties [51, 52, 100]. In this study, the anti-inflammatory properties of 9, 12-octadecadienoic acid (Z, Z)- is a favourable property because it could reduce the inflammation symptoms caused by cellulitis.

Apart from that, n-hexadecanoic acid, octadecanoic acid, 9,12-octadecadienoic acid (Z, Z)- and neophytadiene were identified as the ubiquitous phytochemicals found in the extracts of *C. alata* except for REA maceration and SEA Soxhlet. Both extracts were found deficient in either one phytochemical from the list. Relatively higher concentrations of n-hexadecanoic acid were detected in REA Soxhlet with a peak area of 34.4%, followed by SEA Soxhlet (28.7%) and SEA maceration (12.28%). n-Hexadecanoic acid is also known as palmitic acid, which is the most common saturated fatty acid found in animals, plants and microorganisms with antibacterial, anti-inflammatory, and antioxidants properties [47, 101]. Meanwhile, the highest octadecanoic acid concentration was recorded in SEA Soxhlet with a peak area of 6.68%, followed by REA Soxhlet (3.16%) and SEA maceration (3.01%). Octadecanoic acid is one of the commonest saturated fatty acids and is commonly known by the name stearic acid. It exists as a glycerol ester and is found abundantly in most animals (up to 30%) and plant fats (typically < 5%) [102]. It was reported to exhibit antibacterial, antifungal and antitumour activities [61].

The REA maceration extract was devoid of n-hexadecanoic acid and octadecanoic acid, but it did contain 22.18% of hexadecanoic acid, methyl ester, which was found explicitly in the root extracts of *C. alata*. The REA Soxhlet also contained about 5.44% of hexadecanoic acid, methyl ester. The methyl ester of fatty acid has been reported to exhibit antioxidant and potent antimicrobial activities against Gram-positive and Gram-negative bacteria [48, 103]. A study showed that hexadecanoic acid, methyl ester exhibited antibacterial potency against the clinical strain of *S. aureus*, *Pseudomonas aeruginosa* and *Klebsiella pneumoniae* [99]. The antimicrobial activity of fatty acids is regulated by their structures, morphologies, carbon-chain length functions, and the presence, number, positioning, and orientation of double bonds [104]. Many organisms rely on fatty acid methyl ester as a defence mechanism against bacterial infection, with the mode of action being the bacteria’s cell membrane. In addition, the fatty acid methyl ester also interferes with cellular energy production, inhibits enzyme activity, and causes direct bacterial cell lysis [105]. The safety and activity of the fatty acid methyl ester make it a promising antimicrobial agent.

Although the phytochemicals have been previously identified and characterized in the extracts, 56 phytochemicals whose biological properties and functions remain unknown. Some of these phytochemicals occurred in relatively higher concentrations in the extracts. For example, 3-propylglutaric acid (3.04%) in SEA Soxhlet extract; 4,22-cholestadien-3-one (7.73%) and γ-sitostenone (13.21%) in SEA maceration extract; henicosanal (13.60%), methyl stearate (5.96%) and hexadecanal (4.83%) in REA maceration extract; and methyl 10-trans,12-cis-octadecadienoate (6.76%) and heptadecanolide (6.38%) in REA Soxhlet extract. The phytochemicals that contributed to the antimicrobial, anti-inflammatory and antioxidant properties of *C. alata* in this study should be further investigated before formulating an effective natural drug against the cellulitis causing agent, *S. aureus*.

## Conclusion

The ethyl acetate extracts of *C. alata* exhibited strong antimicrobial activities against the clinical strain of *S. aureus*. The extracts from Soxhlet extraction also exhibited stronger antimicrobial activities compared to maceration extraction. REA Soxhlet extract showed significant inhibition effects towards *S. aureus*, followed by SEA Soxhlet, SEA maceration and REA maceration extract of *C. alata*. Extracts derived from n-hexane, ethyl acetate and ethanol can be utilised as the bactericidal agents (MBC/MIC ≤ 4), except for LHex Soxhlet and LEA maceration extracts. Different elongation patterns of bacterial lag phases and reduction in growth rate were observed during the growth curve analysis. There was a significant regression extension (*p* < 0.06, *p* = 0.00003) of the lag phase for 2 to 6 h after the extract treatment with the increase of extract concentration. Based on the GC–MS analysis, a total of 88 phytochemicals that constitute phenolics, steroids, fatty acids, alcoholics, esters, and alkane hydrocarbons were detected, 32 of which were previously characterized for their antimicrobial, antioxidant, and anti-inflammatory activities.

## Supplementary Information

**Additional file 1.**

**Additional file 2.**

## Data Availability

The data generated or analysed during this study are included in this published article. [Supplementary 1] Temperature, rotations speed, pressure designed for rotary evaporation for each respective extraction solvents. [Supplementary 2] GC–MS chromatogram of REA maceration, REA Soxhlet, SEA maceration, and SEA Soxhlet extract.

## Abbreviations

Hex: N-Hexane
EA: Ethyl acetate
EtOH: Undenatured absolute ethanol
dH_2_O: Sterile distilled water
L: Leave
R: Root
S: Stem
