# Supplementary material for: In vitro antimicrobial efficacy of Cassia alata (Linn.) leaves, stem, and root extracts against cellulitis causative agent Staphylococcus aureus
Source: BMC Complement Med Ther. 2023 Mar 18;23:85. doi: 10.1186/s12906-023-03914-z (PMC10024395; doi:10.1186/s12906-023-03914-z)
Supplement: Supplementary file 1 — Additional file 1. [file 12906_2023_3914_MOESM1_ESM.docx]

**Supplementary Table 1** Temperature, rotations speed, pressure designed for rotary evaporation for each respective extraction solvents.

| **Types of extraction solvent** | **Temperature**  (°C) | **Rotation speed per min**  (rpm) | **Pressure**  (mbar) |
| --- | --- | --- | --- |
| n-Hexane (Hex) | 40 | 80 | <8 |
| Ethyl acetate (EA) | 40 | 80 | <8 |
| Undenatured absolute ethanol (EtOH) | 45 | 80 | <8 |
| Sterile distilled water (dH_2_O) | 50 | 200 | <8 |
