# Supplementary material for: In vitro antimicrobial efficacy of Cassia alata (Linn.) leaves, stem, and root extracts against cellulitis causative agent Staphylococcus aureus
Source: BMC Complement Med Ther. 2023 Mar 18;23:85. doi: 10.1186/s12906-023-03914-z (PMC10024395; doi:10.1186/s12906-023-03914-z)
Supplement: Supplementary file 2 — Additional file 2. [file 12906_2023_3914_MOESM2_ESM.pdf]

Root ethyl acetate (REA) maceration extract

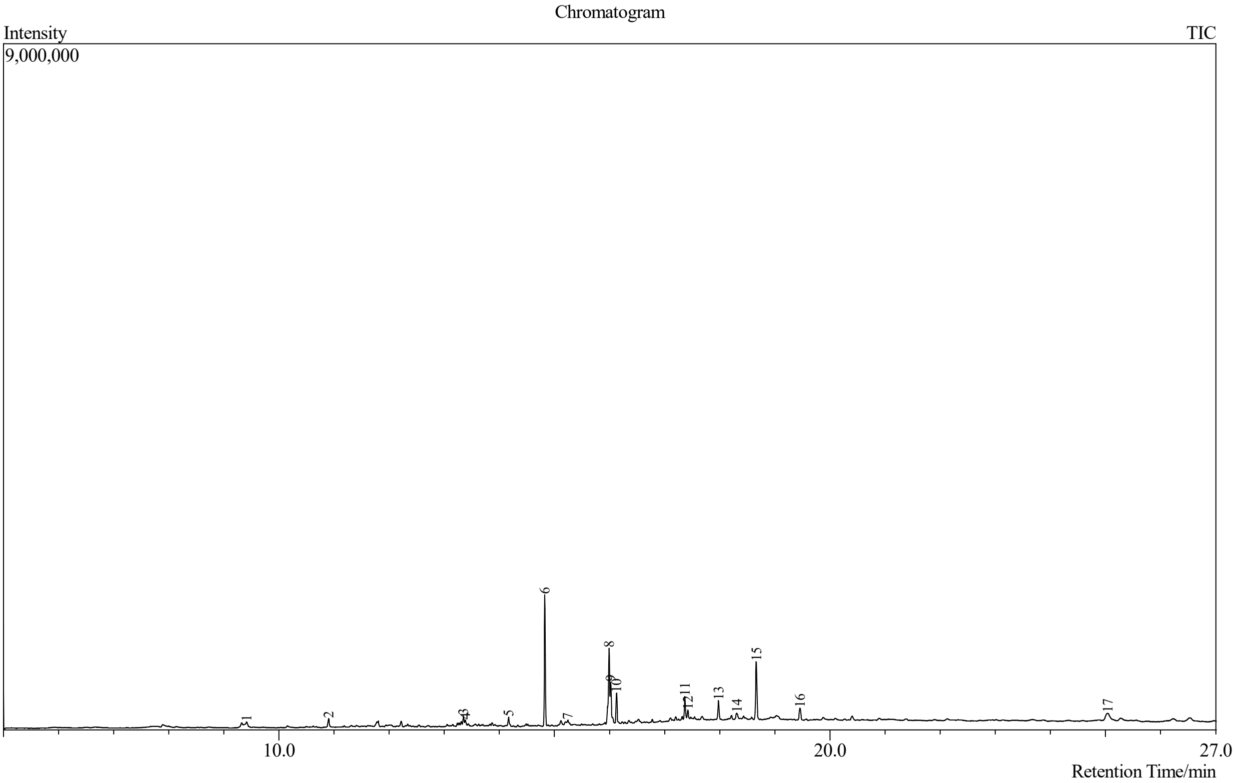

Peak Report TIC

Peak#	R.Time	Area	Area%	Height	Height%	Name
1	9.415	201495	2.12	63838	1.07	Cyclohexasiloxane, dodecamethyl-
2	10.903	179457	1.89	108345	1.81	3-Isopropoxy-1,1,1,7,7,7-hexamethyl-3,5,5-t
3	13.350	216360	2.28	133895	2.24	1-Nonanol, 4,8-dimethyl-
4	13.385	128506	1.35	78639	1.31	13-Tetradecynoic acid, methyl ester
5	14.169	187539	1.98	116402	1.94	Neophytadiene
6	14.826	2105455	22.18	1722181	28.75	Hexadecanoic acid, methyl ester
7	15.244	138476	1.46	63536	1.06	cis-1,2-Cyclododecanediol
8	15.991	1705156	17.97	994559	16.60	11-Octadecenoic acid, methyl ester
9	16.021	812355	8.56	547943	9.15	9-Octadecenoic acid (Z)-, methyl ester
10	16.128	565465	5.96	398768	6.66	Methyl stearate
11	17.367	458626	4.83	305143	5.09	Hexadecanal
12	17.421	210403	2.22	128825	2.15	E-11-Hexadecenal
13	17.976	368894	3.89	246701	4.12	Hexadecanal
14	18.307	187708	1.98	76425	1.28	7-Hexadecenal, (Z)-
15	18.662	1290959	13.60	760809	12.70	Henicosanal
16	19.456	305282	3.22	157960	2.64	Eicosanal-
17	25.040	429089	4.52	86480	1.44	4a,5-Dimethyl-3-(prop-1-en-2-yl)-1,2,3,4,4a,
		9491225	100.00	5990449	100.00	

Root ethyl acetate (REA) Soxhlet extract

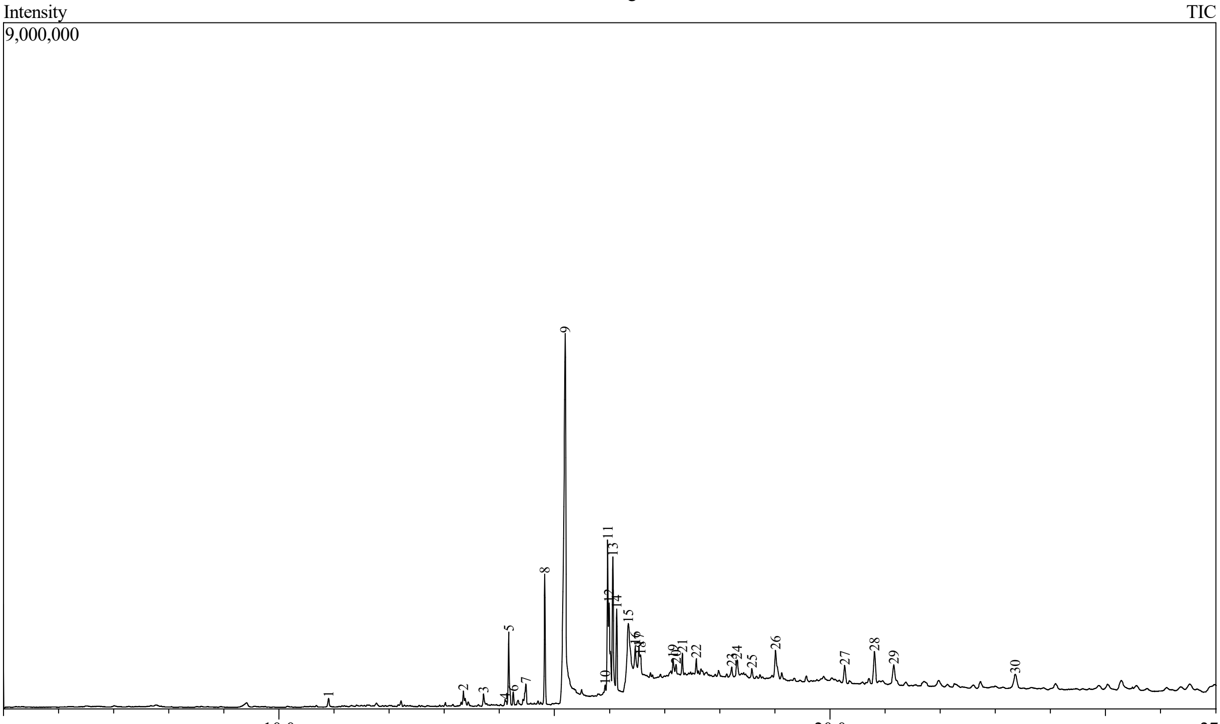

Peak Report TIC

Peak#	R.Time	Area	Area%	Height	Height%	Name
1	10.901	162942	0.41	104114	0.55	3-Isopropoxy-1,1,1,7,7,7-hexamethyl-3,5,5-tı
2	13.349	243421	0.62	178731	0.94	1-Dodecanol, 3,7,11-trimethyl-
3	13.714	227715	0.58	147465	0.77	Tetradecanoic acid
4	14.100	167025	0.42	69723	0.37	2-Hexadecene, 3,7,11,15-tetramethyl-, [R-[R
5	14.170	1412358	3.57	960268	5.03	Neophytadiene
6	14.253	225836	0.57	174964	0.92	2-Pentadecanone, 6,10,14-trimethyl-
7	14.481	508191	1.29	267591	1.40	3,7,11,15-Tetramethyl-2-hexadecen-1-ol
8	14.825	2151171	5.44	1704764	8.93	Hexadecanoic acid, methyl ester
9	15.195	13570274	34.34	4768574	24.98	n-Hexadecanoic acid
10	15.919	122891	0.31	100710	0.53	Pentadecafluorooctanoic acid, octadecyl ester
11	15.965	2673075	6.76	1996620	10.46	Methyl 10-trans,12-cis-octadecadienoate
12	15.991	2022086	5.12	1168795	6.12	9-Octadecenoic acid (Z)-, methyl ester
13	16.059	2632376	6.66	1766952	9.26	Phytol
14	16.129	1416985	3.59	1077339	5.64	Methyl stearate
15	16.341	3979585	10.07	835233	4.38	9,12-Octadecadienoic acid (Z,Z)-
16	16.466	1249888	3.16	463480	2.43	Octadecanoic acid
17	16.532	781613	1.98	404079	2.12	2-Piperidinoethyl p-chlorobenzoate
18	16.565	410529	1.04	270754	1.42	Oxacycloheptadec-8-en-2-one, (8Z)-
19	17.152	340258	0.86	198859	1.04	n-Heptadecanol-1
20	17.205	174342	0.44	127118	0.67	Glycidyl palmitate
21	17.321	373588	0.95	282444	1.48	Methyl 18-methylnonadecanoate
22	17.574	274029	0.69	198244	1.04	4,8,12,16-Tetramethylheptadecan-4-olide
23	18.216	171404	0.43	112079	0.59	1-Monopalmitin, 2TMS derivative
24	18.316	444133	1.12	207751	1.09	7-Hexadecenal, (Z)-
25	18.583	189428	0.48	118033	0.62	Docosanoic acid, methyl ester
26	19.012	977159	2.47	359466	1.88	Heptadecanolide
27	20.267	505670	1.28	231721	1.21	Tetracosanoic acid, methyl ester
28	20.807	1009814	2.56	414749	2.17	Heptadecanolide
29	21.158	563867	1.43	223550	1.17	Squalene
30	23.361	535268	1.35	155729	0.82	Heptadecanolide
		39516921	100.00	19089899	100.00	

Stem ethyl acetate (SEA) maceration extract

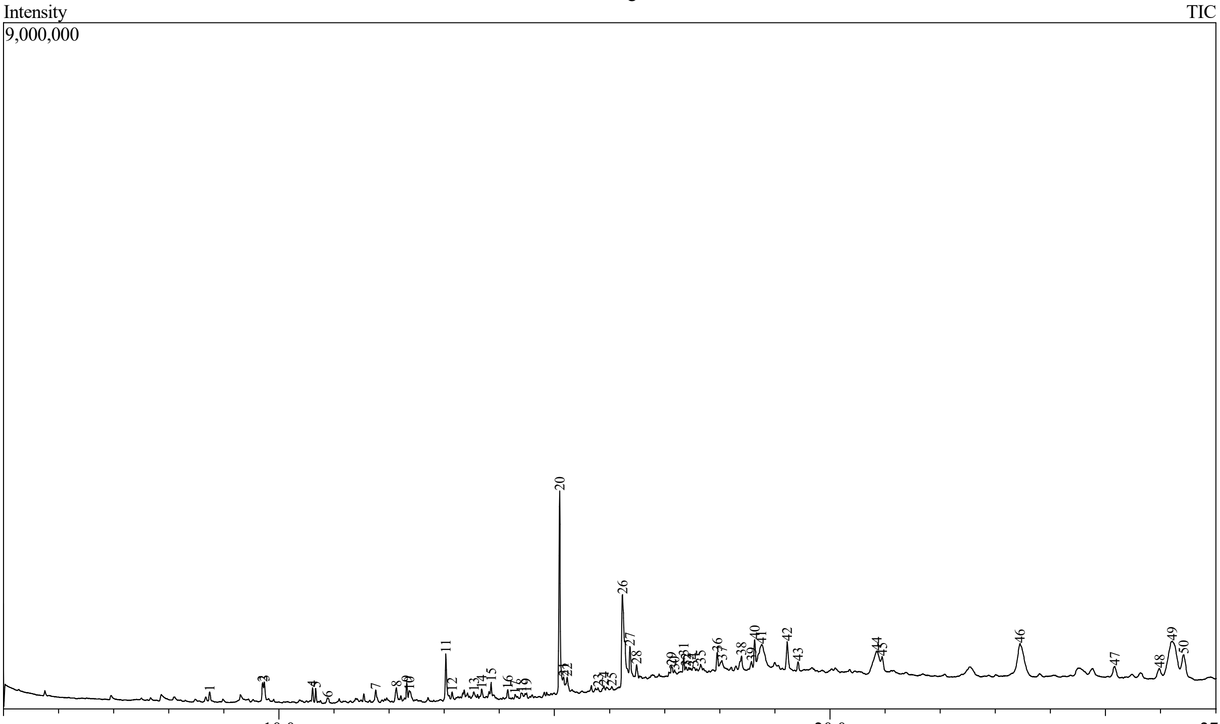

Peak Report TIC

Peak#	R.Time	Area	Area%	Height	Height%	Name
1	8.744	194415	0.51	119460	0.96	Dodecane
2	9.705	396384	1.04	234349	1.87	Caprolactam
3	9.736	378369	1.00	238541	1.91	Tridecane
4	10.611	231729	0.61	180281	1.44	1-Tetradecene
5	10.668	238777	0.63	181642	1.45	Tetradecane
6	10.883	163293	0.43	61976	0.50	Cycloheptasiloxane, tetradecamethyl-
7	11.757	289291	0.76	144901	1.16	Phenol, 3,5-bis(1,1-dimethylethyl)-
8	12.130	406435	1.07	177163	1.42	Eicosanoic acid
9	12.321	446098	1.18	240254	1.92	1-Pentadecene
10	12.367	433185	1.14	138208	1.11	Octadecane
11	13.030	1090439	2.87	601963	4.81	Benzophenone
12	13.144	174836	0.46	102451	0.82	Hexadecane, 2,6,10,14-tetramethyl-
13	13.538	252790	0.67	82121	0.66	Chloroacetic acid, pentadecyl ester
14	13.681	236777	0.62	116404	0.93	Eicosanoic acid
15	13.850	242960	0.64	185121	1.48	n-Nonadecanol-1
16	14.153	168502	0.44	110060	0.88	Neophytadiene
17	14.285	165417	0.44	58882	0.47	Benzophenone, 2,4,6-trimethyl-
18	14.400	207665	0.55	74729	0.60	Pentadecanoic acid
19	14.494	201884	0.53	70671	0.57	1,2-Benzenedicarboxylic acid, bis(2-methylp
20	15.094	4658394	12.28	2659723	21.27	n-Hexadecanoic acid
21	15.165	376010	0.99	205621	1.64	Dibutyl phthalate
22	15.230	515859	1.36	191035	1.53	n-Nonadecanol-1
23	15.791	157127	0.41	48288	0.39	Octadecanoic acid, 2-propenyl ester
24	15.898	164617	0.43	69550	0.56	Tetratetracontane
25	16.040	219902	0.58	48801	0.39	Hentriacontane-10,14,16-trione, TMS derivat
26	16.233	4421091	11.65	1205410	9.64	9,12-Octadecadienoic acid (Z,Z)-
27	16.372	1143484	3.01	471639	3.77	Octadecanoic acid
28	16.490	431992	1.14	186443	1.49	Trifluoroacetic acid, pentadecyl ester
29	17.126	277820	0.73	125433	1.00	1-Eicosanol
30	17.175	289374	0.76	63929	0.51	Glycidyl palmitate
31	17.345	322697	0.85	219290	1.75	Henicosanal
32	17.388	172351	0.45	78138	0.62	Bicyclo[3.1.1]heptan-3-ol, 6,6-dimethyl-2-m
33	17.435	211068	0.56	58004	0.46	Tetrapentacontane
34	17.523	154719	0.41	56507	0.45	Cycloheptanol, 2-methylene
35	17.653	191160	0.50	76317	0.61	Eicosyl trifluoroacetate

Peak#	R.Time	Area	Area%	Height	Height%	Name
36	17.954	410859	1.08	236498	1.89	Eicosanal-
37	18.040	400491	1.06	104449	0.84	1(3H)-Isobenzofuranone, 5-hydroxy-3-[(4-hy
38	18.394	530804	1.40	181556	1.45	Tetradecane, 1-chloro-
39	18.575	197890	0.52	97483	0.78	Zeranol
40	18.633	715220	1.88	373679	2.99	Henicosanal
41	18.760	2170950	5.72	296206	2.37	Stigmasterol
42	19.225	827358	2.18	363988	2.91	Triphenylphosphine oxide
43	19.421	226458	0.60	120160	0.96	Eicosanal-
44	20.849	1907462	5.03	266470	2.13	.gamma.-Sitosterol
45	20.947	516871	1.36	188507	1.51	Decanedioic acid, bis(2-ethylhexyl) ester
46	23.448	2932255	7.73	383833	3.07	4,22-Cholestadien-3-one
47	25.165	523852	1.38	134030	1.07	Stigmasta-5,22-dien-3-ol, acetate, (3.beta.,22.
48	25.978	447419	1.18	108809	0.87	Cholesta-4,6-dien-3-ol, (3.beta.)-
49	26.209	5012306	13.21	471727	3.77	.gamma.-Sitostenone
50	26.416	1498214	3.95	293392	2.35	.beta.-Sitosterol acetate
		37945320	100.00	12504092	100.00	

Stem ethyl acetate (SEA) Soxhlet extract

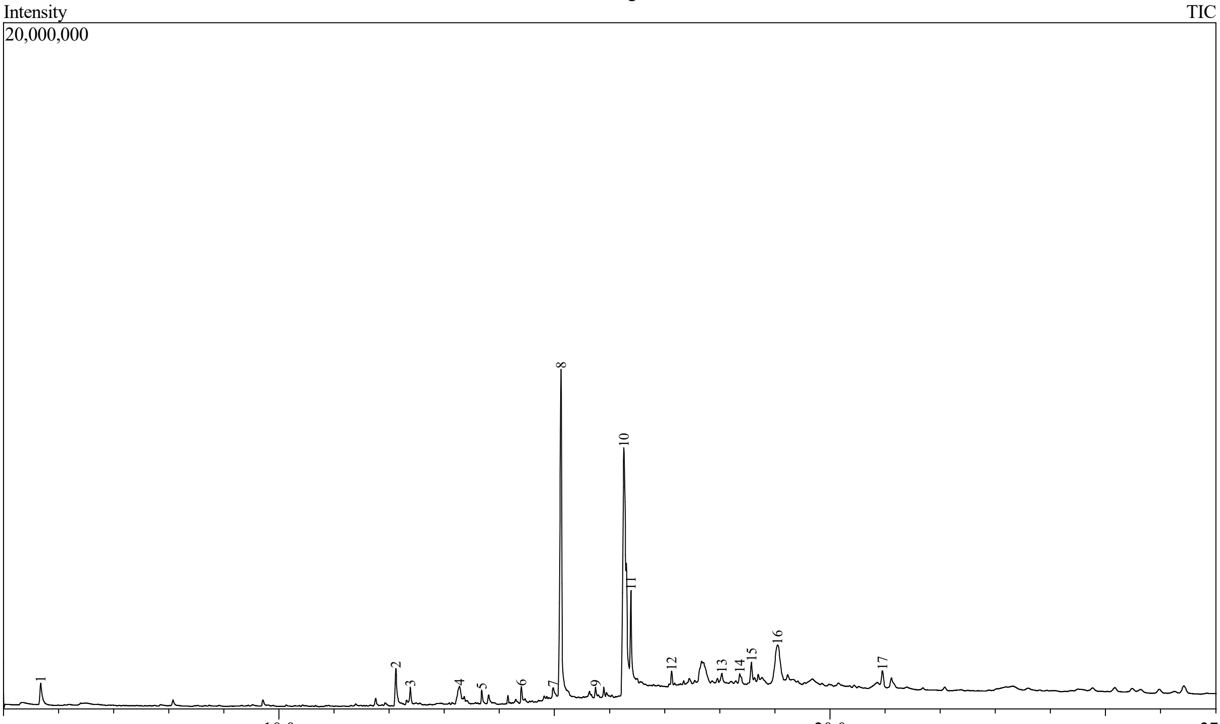

Peak Report TIC

Peak#	R.Time	Area	Area%	Height	Height%	Name
1	5.677	1608969	2.17	607290	2.31	Ethane, 1,1,2,2-tetrachloro-
2	12.122	2252682	3.04	1059908	4.04	3-Propylglutaric acid
3	12.385	719693	0.97	452910	1.73	1H-2-Benzopyran-1-one, 3,4-dihydro-8-hydr
4	13.277	1993306	2.69	477258	1.82	D-Fructose, 3-O-methyl-
5	13.681	634232	0.85	395781	1.51	Tetradecanoic acid
6	14.400	681936	0.92	443306	1.69	Pentadecanoic acid
7	14.976	737839	0.99	284947	1.09	Palmitoleic acid
8	15.120	21364380	28.79	9465076	36.08	n-Hexadecanoic acid
9	15.746	436853	0.59	285230	1.09	Heptadecanoic acid
10	16.261	28198737	38.00	7108872	27.10	9,12-Octadecadienoic acid (Z,Z)-
11	16.388	4955629	6.68	2697405	10.28	Octadecanoic acid
12	17.127	578752	0.78	387070	1.48	1-Eicosanol
13	18.037	429070	0.58	225725	0.86	9,10-Anthracenedione, 1,8-dihydroxy-3-metl
14	18.363	803431	1.08	282816	1.08	Behenic alcohol
15	18.575	1196512	1.61	582318	2.22	Zeranol
16	19.047	6573848	8.86	1045623	3.99	Tetracontane
17	20.953	1034008	1.39	433566	1.65	Decanedioic acid, bis(2-ethylhexyl) ester
		74199877	100.00	26235101	100.00	
